# Sepsis-induced thrombocytopenia: early prediction and modifiable mortality

**DOI:** 10.1186/cc9862

**Published:** 2011-03-11

**Authors:** G Georgiev, S Milanov, V Todorova, L Kozarov, M Milanov

**Affiliations:** 1 Pirogov Emergency Institute, Sofia, Bulgaria

## Introduction

Thrombocytopenia is a common problem in the ICU, considered to be associated with increased morbidity and mortality. Risk factors for sepsis-induced thrombocytopenia have not been yet specified. Our study focuses on its development and consequences in the general ICU.

## Methods

A prospective observational study was conducted including all cases of sepsis for a 2-year period. Data on demographics, primary diagnosis and source of infection, current infectious pathogens, presence/severity of shock and outcome were cross-tabulated according to the presence and severity of thrombocytopenia. Effects of immunotherapy and substitution with thrombocyte concentrate on outcome were also tested. Analyses of disease prescription, length of ICU stay (LOS), severity and dynamics of organ dysfunction/disease/systemic inflammation (serum creatinine levels, SOFA, SAPS II, SIRS, lung injury score) for each group of patients was performed.

## Results

The study included 118 patients with thrombocytopenia of variable severity (39.33% out of 300 septic patients). The following independent prognostic factors for supervening thrombocytopenia (reported with respective RR and 95% CI) were identified: platelet count <150 g/l - prescription >48 hours (1.31; 1.02 to 2.67), SOFA score on inclusion >6 (1.36; 1.02 to 1.78), ΔSOFA >5 (2.77; 2.17 to 3.50), initial SAPS II exp. score >5.5 (1.39; 1.04 to 1.82), LIS >1.75 (1.56; 1.13 to 2.19), serum creatinine >122 μmol/l (2.38; 1.72 to 3.36), Gram-positive infectious pathogen, especially if Gram-positive co-infection or if concomitant invasive candidiasis (1.44, 1.08 to 1.94; 1.9, 1.33 to 2.46 and 2.60; 1.31 to 3.02), *Streptococcus *spp. infection (2.04; 1.17 to 2.64), disruption of the lower GIT (1.48; 1.06 to 1.97), polytrauma (0.39; 0.22 to 0.65); and platelet count <20 g/l - urosepsis (6.53; 1.76 to 16.96), soft tissue infection (9.87; 2.75 to 22.07), initial SAPS II exp. score >6.2 (4.90; 2.02 to 11.71), SOFA score on inclusion >8 (6.52; 2.68 to 15.04), female sex (1.74; 1.13 to 2.26). Mortality was significantly higher for the thrombocytopenic patients (66.95% vs. 41.76%, *P *= 0.000), except for those who underwent specific therapy (37.5%, *P *= 0.000).

## Conclusions

New insight was gained into the prediction of imminent sepsis-induced thrombocytopenia. Applied immunotherapy and substitution therapy for the most severely but early identified thrombocytopenic patients contributes to the inadvertently reduced mortality within this group.

